# Molecular Dynamic Simulation Analysis on the Inclusion Complexation of Plumbagin with β-Cyclodextrin Derivatives in Aqueous Solution

**DOI:** 10.3390/molecules26226784

**Published:** 2021-11-10

**Authors:** Kulpavee Jitapunkul, Pisanu Toochinda, Luckhana Lawtrakul

**Affiliations:** School of Bio-Chemical Engineering and Technology, Sirindhorn International Institute of Technology, Thammasat University, Bangkok 12120, Pathum Thani, Thailand; kulpavee.work@gmail.com (K.J.); pisanu@siit.tu.ac.th (P.T.)

**Keywords:** inclusion complexes, β-cyclodextrin, 2-*O*-monohydroxypropyl-β-cyclodextrin, dimethyl-β-cyclodextrin, plumbagin, molecular dynamics simulations

## Abstract

Stable encapsulation of medically active compounds can lead to longer storage life and facilitate the slow-release mechanism. In this work, the dynamic and molecular interactions between plumbagin molecule with β-cyclodextrin (BCD) and its two derivatives, which are dimethyl-β-cyclodextrin (MBCD), and 2-*O*-monohydroxypropyl-β-cyclodextrin (HPBCD) were investigated. Molecular dynamics simulations (MD) with GLYCAM-06 and AMBER force fields were used to simulate the inclusion complex systems under storage temperature (4 °C) in an aqueous solution. The simulation results suggested that HPBCD is the best encapsulation agent to produce stable host–guest binding with plumbagin. Moreover, the observation of the plumbagin dynamic inside the binding cavity revealed that it tends to orient the methyl group toward the wider rim of HPBCD. Therefore, HPBCD is a decent candidate for the preservation of plumbagin with a promising longer storage life and presents the opportunity to facilitate the slow-release mechanism.

## 1. Introduction

Plumbagin, or 2-methyljuglone, is an essential plant-based naphthoquinone that has been extensively used as a medicinal compound in Asian countries [1]. It exhibits several influential biological effects such as antioxidant, anti-inflammatory, anticancer, antibacterial, and antifungal activities [2]. Moreover, cytotoxic activity against lung and breast carcinoma has been reported in several studies [3,4,5]. Unfortunately, the cytotoxicity of the medicinal compound can also affect healthy cells [6]. Moreover, plumbagin has low water solubility, bioavailability, and melting point, which lead to the compound’s instability [7,8]. Recently researchers have reported that nano-encapsulation of plumbagin with β-cyclodextrin (BCD) can reduce toxicity and enhance pharmaceutical efficacy [9,10]. Our works have also reported the use of BCD and its derivatives to enhance the stability and aqueous solubility of plumbagin [11,12].

Cyclodextrins are cyclic oligosaccharides with hydrophobic inner cavities and hydrophilic surfaces. Since 1953, BCD has been one of the well-known encapsulation agents for biologically active compounds. For simplicity, they usually are described as a truncated cone with a wider rim and narrow rim comprised of secondary and primary hydroxyl groups, respectively. The BCD inclusion complex can provide higher thermal stability, slower release rate, and resistance to α-amylase in saliva for various active compounds. However, water solubility has not been fairly improved by encapsulation with BCD due to its strong crystal lattice energy. Scientific studies have suggested that the substitution of primary or secondary hydroxyl groups with other functional groups can lead to higher water solubility [13,14,15,16]. Two BCD derivatives of interest are dimethyl-β-cyclodextrin (MBCD) and 2-*O*-monohydroxypropyl-β-cyclodextrin (HPBCD), which have been reported to substantially improve the solubility of the inclusion complex [17,18].

Our previous experimental and computational studies revealed that the preservation of plumbagin can be intensely improved by encapsulation with BCD under an affordable price scheme. Furthermore, the molecular docking and semi-empirical calculations of BCD and its two derivatives (MBCD and HPBCD) with plumbagin suggested that the inclusion complexes are possible. There were two potential binding conformations, so-called conformation-I and conformation-II which have been defined by the position of plumbagin’s methyl group inside the bound cavity. The methyl group was located near the wider rim of the truncated cone in conformation-I and near the narrow rim in conformation-II [10,12]. Nevertheless, the intermolecular interactions and dynamics of plumbagin and β-cyclodextrins (BCDs) inclusion complexes were not fully investigated, especially not under an aqueous solution with thermal effects.

Therefore, in this work, molecular dynamics simulations (MD) were performed to investigate the structural dynamic behavior of the plumbagin–BCDs inclusion complexes in an aqueous solution under the storage temperature (4 °C). The free binding energy and binding–releasing pathway of inclusion complexes were observed to find the finest host for plumbagin to enhance its stability, which could be supportive information on the encapsulation and transport processes of plumbagin.

## 2. Results

### 2.1. Solvated Inclusion Complexes Equilibrium and Stability

The total system energy of all equilibrated inclusion complexes in an aqueous solution under a storage temperature of 4 °C was observed throughout 200 ns MD simulations. For both conformations (I and II) of plumbagin–BCD and plumbagin–HPBCD inclusion complexes (BCD-I, BCD-II, HPBCD-I, and HPBCD-II), the energies were steady at −7000 kcal/mol. For plumbagin–MBCD inclusion complexes, in both conformations (MBCD-I and MBCD-II), the energies were steady at −9000 kcal/mol. Thus, all solvated inclusion complexes fully reached equilibrium and were considered to be stable isothermal systems.

**Figure 1:**
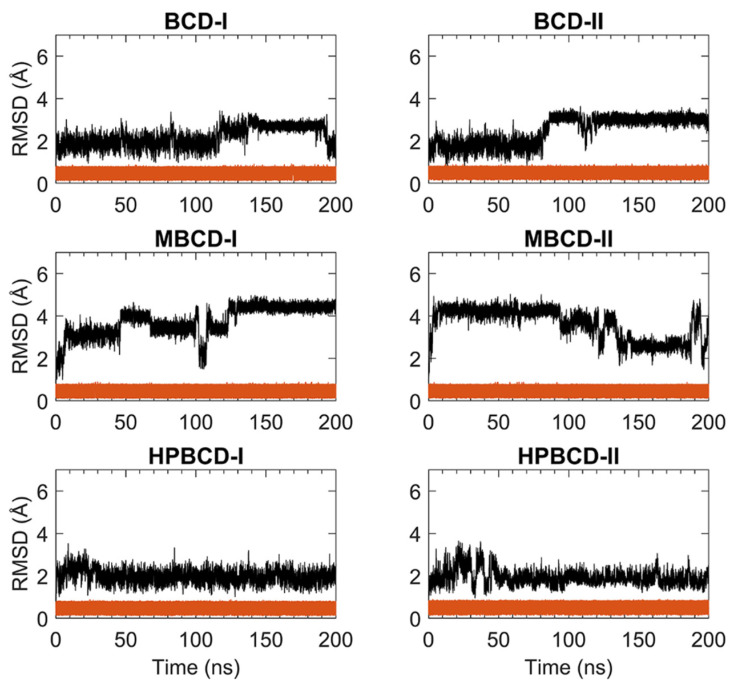
RMSD plots of plumbagin and BCDs during 200 ns MD simulations. The red and black lines represent the RMSD of plumbagin and BCDs, respectively.

For stability analysis of plumbagin and hosts molecules, the all-atom root-mean-square deviation (RMSD) of plumbagin and BCDs were plotted using red and black lines, respectively, as shown in Figure 1. The all-atom RMSD of the host’s molecules presented higher deviation than plumbagin molecule, as the consequence of the difference in molecular size and chemical structure. Plumbagin is a naphthoquinone that has limited structural flexibility; thus, its structural deviation throughout the whole MD simulation was small and steady (RMSD of 0.1 to 0.9 Å); meanwhile, hosts molecules—which are cyclic oligosaccharides with seven glucose units—lead to a higher degree of structural motions, especially when there are substituted functional groups on the rims of a truncated cone (e.g., MBCD and HPBCD).

The BCD structure of BCD-I was considerably similar to the reference’s orientation with low RMSD values (1.2 to 2.5 Å) during the first 115 ns. Afterward, the structural deviation of BCD started to increase and then re-stabilized within the next 30 ns. This lead to another stable interval with steady RMSD fluctuation between 2.8 and 3.1 Å until 190 ns. Then, they were probably reformed back to the reference’s orientation again, which is indicated by low RMSD. While the BCD structure in BCD-II stayed in the reference’s structure until 82 ns, its RMSD value then increased to be around 3 Å and steady until the end of simulations.

For plumbagin–MBCD inclusion complexes (MBCD-I and MBCD-II), the structural deviation of MBCD molecules was the highest among other inclusion complexes (RMSD ranging from 1.8 to 4.8 Å). The high fluctuation could be the result of additional dynamic motions of substituted methyl groups on both rims of the truncated cone. Nevertheless, several steady intervals were found during 200 ns MD simulations. In MBCD-I, the MBCD structure was gradually deviated until reaching a maximum distance of 3.95 Å within the first 10 ns. Then, there were three short stable intervals, 20 to 40 ns each, until 100 ns, with 2.85 to 4.35 Å fluctuation. Interestingly, the RMSD profile was highly fluctuated, ranging from 1.8 to 4.8 Å during 100 to 130 ns, which could refer to large structural motions within this specific period. However, after 130 ns, the RMSD fluctuation was stabilized within the 4.0 to 4.8 Å range until the end of simulation, which indicated the restoration of MBCD’s structural stability. Inversely, the RMSD of the MBCD molecule in MBCD-II revealed a large structural deviation during the first 90 ns with steady fluctuation (3.9 to 4.6 Å). Then, the deviation of MBCD structure gradually declined during 90 to 130 ns to the minimum distance of 1.9 Å. Afterward, the structural fluctuation was kept steady between 2.5 and 3.1 Å until 185 ns. However, the high degree of structural deviation arose again toward the end of simulation, with RMSD fluctuating between 1.8 to 4.8 Å. This was an interesting structural motion of MBCD from two inclusion complex conformations because the RMSD profiles appeared to be in reversed trend.

The finest dynamics system occurred in both conformations of plumbagin–HPBCD inclusion complexes (HPBCD-I and HPBCD-II). However, the slight RMSD fluctuation (1.0 to 3 Å) can still be found during the first 30 and 50 ns for HPBCD-I and HPBCD-II, respectively. These structural deviations at the beginning of simulations might have come from motions of a hydroxypropyl group attached to the wider rim of HPBCD. The RMSD profiles of HPBCD were noticeably steady (RMSD of 1.0 to 2.2 Å) during the last 170 and 150 ns of the simulation time for HPBCD-I and HPBCD-II, respectively.

### 2.2. Dynamics Behavior of Inclusion Complexes

**Figure 2:**
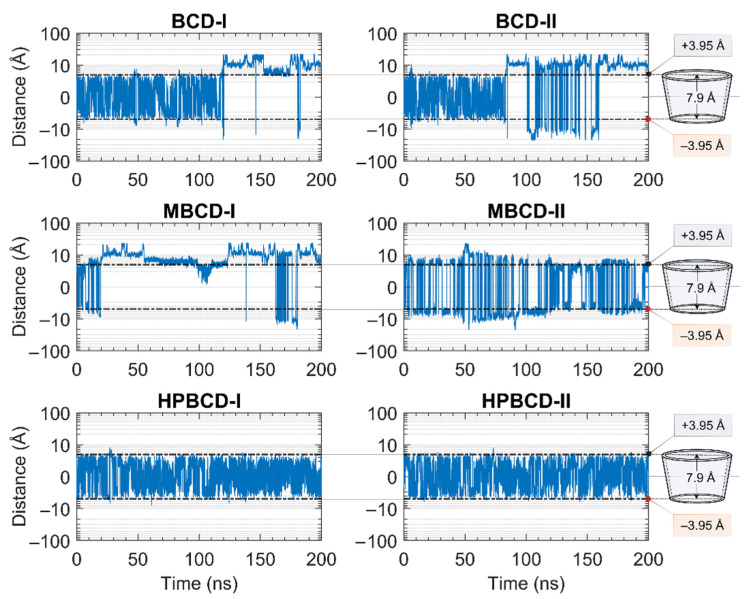
Plots of the distance between the center of mass of plumbagin and BCDs during 200 ns MD simulations. The y-axis of all subplots is presented in a logarithmic scale for a clearer presentation. The black horizontal dashed lines indicate the distances from the center of mass of BCD core structure to the wider rim and narrow rim with the distances labeled as +3.95 and −3.95 Å, respectively. The truncated cone diagrams on the right represent the total vertical dimension of BCD’s core structures, without substituted functional groups.

The stability of inclusion complexes was extensively analyzed based on the structural deviation of the BCDs molecule the using all-atoms RMSD profiles in the previous section. However, the RMSD value is not a sufficient parameter to fully represent plumbagin movement inside the simulated space. Therefore, the distance between the centers of mass of plumbagin and BCDs were measured to investigate the position of plumbagin during MD simulations, as shown in Figure 2. However, to avoid defects in calculation due to asymmetrical structure, the substituted functional groups of BCD derivatives were not taken into consideration. The center of mass of BCD’s core structure was considered to be the origin; thus, positive and negative distances indicated whether the plumbagin molecule was located above or below that origin. The black horizontal dashed lines on each subplot represent half of the vertical distance of BCD core structure in the positive and negative directions. Therefore, the positive and negative distances above or below these horizontal lines indicate the release of the plumbagin molecule from BCDs inner cavity.

The plumbagin molecule bounced upward and downward within the BCD cavity for the first 115 and 82 ns in BCD-I and BCD-II conformations, respectively. Then, the plumbagin molecule started to migrate out from the binding cavity with several attempts to migrate back inside the cavity space. However, the plumbagin could not be stabilized inside the inner cavity of BCD anymore, which could be a consequence of structural deviation of BCD, as mentioned in the stability analysis. 

Similarly, the relationship between the structural deviation of MBCD and dynamics of plumbagin could be identified as follows. For MBCD-I conformation, the MBCD structure deviation could be roughly divided into three stable intervals during the first 100 ns, which also affects the movement of plumbagin in the same way. In other words, plumbagin bounced up and down inside the cavity during the first 20 ns and then migrated out and kept a constant distance with MBCD until 50 ns. Afterward, it shifted back and stayed closer to the MBCD again until 100 ns. During 100 and 130 ns, in which RMSD indicated the high structural fluctuation of MBCD, plumbagin was attracted back into MBCD cavity. However, the highly fluctuated movement was found after 130 ns and the plumbagin mostly stayed away from MBCD inner cavity, which corresponds to the final stable interval of MBCD structural deviation. For MBCD-II conformation, the relationship between MBCD structural deviation and plumbagin movement interpretation is difficult due to the high fluctuation of plumbagin movement: it intensively bounced upward and downward both inside and outside the MBCD cavity. Thus, the trend of plumbagin dynamics could not be clearly seen. However, the distance profile could be divided into three main intervals with different plumbagin movements, and they are roughly comparable to the RMSD profile of MBCD. In the first interval, the highest fluctuation of plumbagin movement could be observed during the first 130 ns, which represented the interchange between its bound and released states. The second interval was defined between 130 and 185 ns and the fluctuation of plumbagin movement was reduced, which resulted in a bouncing motion within MBCD cavity only. In the third interval, the fluctuation of plumbagin movement increased until the end of the simulation, which represented the revival of interchange between bound and released states.

The movement of plumbagin in HPBCD-I and HPBCD-II conformations was stable, as expected, because the high stability of HPBCD structural deviation was previously observed. Moreover, the plumbagin molecule bounced up and down inside the binding cavity with no evidence of migration to the solvated space.

Even though the structural movement of BCDs and dynamics of plumbagin can be predicted using all-atom RMSD and distance profile, the visualization of inclusion complexes is also important for behavior analysis. According to previously analyzed results, there are multiple stable intervals during MD simulations that could be selected for further analysis. After careful consideration based on RMSD profiles and distance profiles, two stable intervals were selected. The first stable interval (15 to 20 ns) was chosen to represent the initial bound state during MD simulations. The second stable interval (145 to 150 ns) was used to represent the dynamics behavior of inclusion complexes during the latter part of simulations.

**Figure 3:**
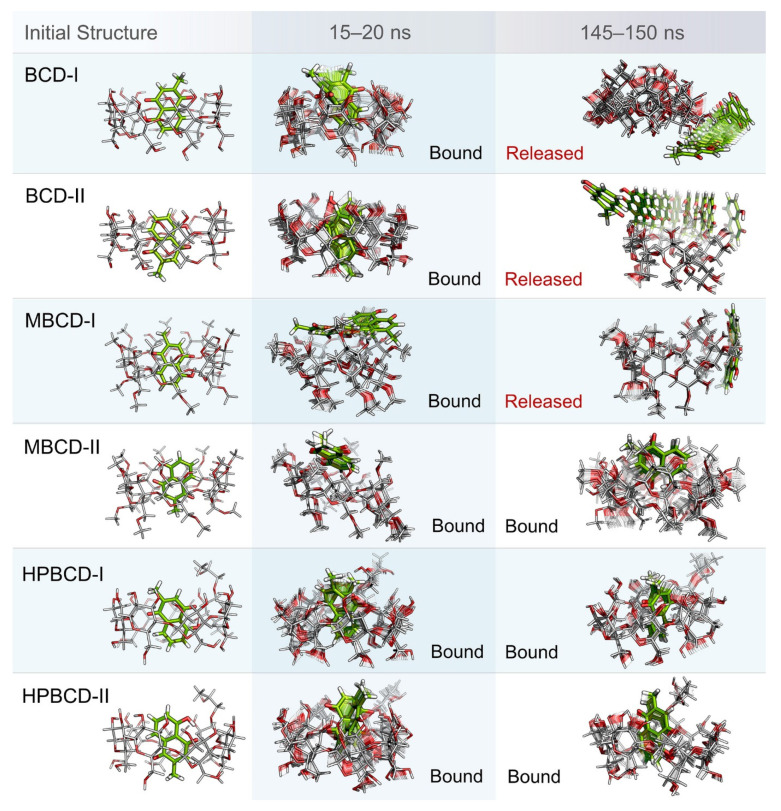
Illustrations of initial inclusion complex structures and dynamic snapshots from two stable intervals. Plumbagin and BCDs molecules are presented as green and light gray stick models, respectively. In both molecules, oxygen and hydrogen atoms are highlighted with red and white color, respectively. For dynamic snapshots, all intermediate frames are presented with the transparent model, only the first and the last frames were illustrated without transparency. Bound and released states are specified according to the positions and motions of plumbagin molecule.

Ten snapshots during the selected stable intervals were illustrated in comparison with the initial structure from structural minimization as shown in Figure 3. During 15 to 20 ns, plumbagin molecule in all inclusion complexes was located inside or near the rim of BCDs cavity, which is consistent with the distance profiles. However, the motion of the plumbagin molecule was elevated during the latter stable interval (145 to 150 ns) in three inclusion complexes, which are the BCD-I, BCD-II, and MBCD-I conformations. Thus, this resulted in the release of a plumbagin molecule from the inner cavity of BCD and MBCD into the solvated space. According to the all-atom RMSD profiles of these three inclusion complexes, the structural deviation of BCD and MBCD was also increased during the latter part of the simulations, which can be used to support the observation from molecular visualization. On the other hand, the plumbagin molecule was not released from the inner cavities of the MBCD-II, HPBCD-I, and HPBCD-II conformations during the latter stable interval. Therefore, these three inclusion complexes should provide a stronger binding between the hosts (BCDs) and the guest (plumbagin) molecule than the previous three systems.

In addition, the visualization of MD snapshots can also reveal the interesting behavior of the plumbagin molecule in each inclusion complex. For the BCD-I conformation, the plumbagin molecule was released from the narrow rim, which was enlarged due to the structural deformation of BCD, and its methyl group was rotated down approximately 90 degrees. For the BCD-II conformation, the alignment of plumbagin was kept at the original orientation, even during the migration from BCD’s inner cavity. For MBCD-I and MBCD-II conformations, the plumbagin molecule floated up to the wider rim of MBCD with changing in orientation by pointing its methyl group toward the side of MBCD cavity. However, after plumbagin was released in MBCD-I conformation, its alignment seemed to be random and clung on the outer surface of MBCD with some weak interaction. For the HPBCD-I conformation, the orientation of plumbagin inside the HPBCD cavity did not change at all. Inversely, the plumbagin molecule flipped its methyl group toward the wider rim of HPBCD in the HPBCD-II conformation, and this meant that plumbagin preferred to orient as conformation-I inside HPBCD’s cavity.

### 2.3. Binding Energies and Intermolecular Interactions

**Figure 4:**
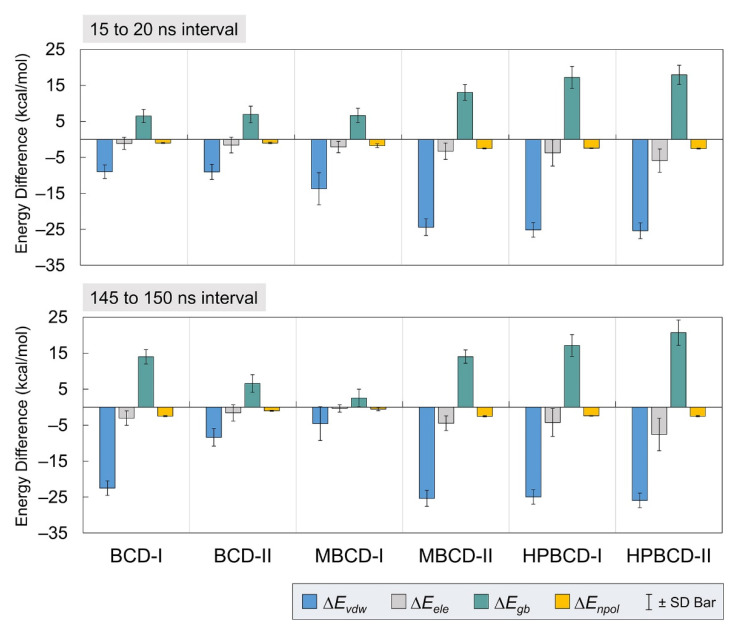
Plots of energetic contributions from two stable intervals. The van der Waals (Δ*E_vdw_*) and electrostatic (Δ*E_ele_*) energies, which contribute to average interaction energy, are presented as blue and gray bars, respectively. The electrostatic (Δ*E_gb_*) and nonpolar (Δ*E_npol_*) energies, which contribute to solvation-free energy, are presented as teal and yellow bars, respectively. The error bar is included for each bar plot to represent the standard deviation values.

To gain more understanding about the binding interactions between BCDs and plumbagin in each inclusion complex, molecular mechanics–generalized born surface area continuum solvation (MM/GBSA) approach [19] was used to estimate the binding energy. The calculations were based on 5,000 frames from the two selected stable intervals (15 to 20 ns and 145 to 150 ns). The binding energy and all energetic contributions were plotted and listed in Figure 4 and Table 1, respectively.

The average interaction energy in the gas phase (Δ*G_gas_*) was the summation of van der Waals (Δ*E_vdw_*) and electrostatic (Δ*E_ele_*) energies. The solvation-free energy in the implicit aqueous phase (Δ*G_sol_*) was the summation of electrostatic (Δ*E_gb_*) and nonpolar (Δ*E_npol_*) energies. Therefore, the total energy difference (Δ*G_Total_*) from the binding was the summation of Δ*G_gas_* and Δ*G_sol_*. Then, the entropy change (*T*Δ*S*) of plumbagin from the host–guest complexation at a storage temperature was subtracted from Δ*G_Total_* to obtain the binding energy (Δ*G_bind(MM/GBSA)_*).

In Figure 4, all inclusion complexes during both stable intervals showed negative van der Waals and electrostatic contributions in gas phase energy; however, they showed small negative electrostatic and highly positive nonpolar contributions in solvation-free energy. Therefore, all inclusion complexes complied with the typical phenomena, which are the favorable interaction energy in the gas phase and unfavorable solvation-free energy. The reason that most systems would have unfavorable solvation-free energy is that this energetic contribution refers to the de-solvation process of bound molecules from solvated space and restoring the binding interface. It was also very clear that the leading contribution for host–guest binding in all plumbagin–BCDs inclusion complexes was van der Waals interactions.

From Table 1, the negative total energy difference and binding energy of all inclusion complexes, during both stable intervals, indicated the favorable host–guest complexations. However, the binding affinity among all inclusion complexes is different, with several interesting perspectives. For the 15 to 20 ns interval, the total energy difference suggests the binding affinity rank without the effect of entropy change, as follows: MBCD-II > HPBCD-II > HPBCD-I > MBCD-I > BCD-II > BCD-I. For the 145 to 150 ns interval, the ranking is MBCD-II > HPBCD-II > HPBCD-I > BCD-I > BCD-II > MBCD-I. Comparison between the results from two stable intervals showed the switching of binding affinity ranking of BCD-I and MBCD-I, which are the inclusion complexes with evidence of plumbagin release. Thus, this leads to one important question, which is that of why the binding affinity between plumbagin and BCD in BCD-I conformation was stronger, with evidence of plumbagin leaving the binding cavity. Then, a hypothesis was proposed as follows: the stronger binding between plumbagin and BCD may occur at the rim, which consists of hydroxyl groups. If this hypothesis is true, this means that the release of plumbagin in BCD-I conformation is not the complete release.

However, the binding affinity analysis, based solely on the total energy difference, was not comprehensive because the entropy effect was excluded. Therefore, the binding affinity based on MM/GBSA binding energy was investigated as well. For 15 to 20 ns, the binding affinity ranking is MBCD-II > MBCD-I > BCD-I > BCD-II > HPBCD-II > HPBCD-I. For 145 to 150 ns, the binding affinity ranking is MBCD-I > BCD-II > BCD-I > MBCD-II > HPBCD-I > HPBCD-II. By considering the entropy change listed in Table 1, there were both positive and negative entropy changes. As generally known, a negative entropy change implies that the system is in high order and positive entropy implies the elevation is in disorder. Therefore, BCD-II, BCD-II, and MBCD-I conformations tend to progress toward disorder, with respect to time, because the entropy changes are positive and elevated, especially in BCD-II and MBCD-I conformations. For the MBCD-II conformation, the entropy change increased from a small negative value to a small positive value, which can suggest that this system is not stable because the motion of molecules in this system tends to generate disorder as well. On the other hand, HPBCD-I and HPBCD-II conformations produced the negative entropy change, which supports the immobility of plumbagin inside HPBCD cavity. Thus, plumbagin–HPBCD inclusion complexes tend to produce the highest stability of host–guest binding, even though the binding energy (Δ*G_bind(MM/GBSA)_*) is not strong compared with others.

To validate the calculated binding energies, the data from other literature have been listed for comparison in Table 2. The results show that the calculated energy difference of inclusion complexes, without entropic contribution, is comparable with published data from molecular docking and semi-empirical PM6 method. However, the reason that the result from HPBCD-II shows a high deviation from the published value was a consequence of the changing of plumbagin alignment to conformation I. Thus, our calculated energy difference of the HPBCD-II conformation during both stable intervals was represented in the inclusion complex conformation I. From this comparison, the calculated results were considered to be reliable.

**Figure 5:**
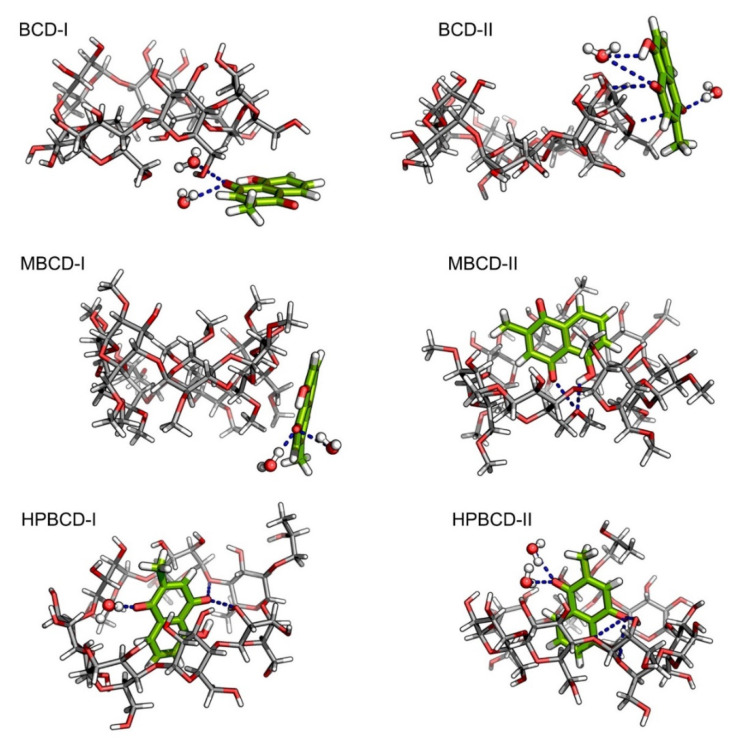
Illustrations of snapshot at 145 ns from all plumbagin–BCDs inclusion complexes. Plumbagin and BCDs molecules are presented as green and light gray stick models, respectively. The bound water molecules are presented as ball and stick models. In all molecules, oxygen and hydrogen atoms are highlighted with red and white colors, respectively. The blue dash lines represent the hydrogen bonds.

Despite the fact that the plumbagin binding behavior can be obtained based on interpretation from binding energy and entropy change, the intermolecular interaction between plumbagin and BCDs is not fully understood. Consequently, the intermolecular bonding of the inclusion complexes was investigated from snapshots during the latter stable intervals (145 to 150 ns) in order to find the key factor that facilitates the binding or release mechanisms. The interaction that could be clearly visualized was hydrogen bonding, which was related to electrostatic contribution, as shown in Figure 4. However, it is not necessary that the hydrogen bond will always be found within the inclusion complexes because the small ligand might be attracted to the BCDs solely on hydrophobic interaction. The representative snapshots (145 ns) from each conformation were illustrated in Figure 5 with hydrogen bonding shown as the blue dash line.

According to our hypothesis about the interaction between plumbagin and BCD in BCD-I conformation, after the detailed visualization, there was no hydrogen bonding between plumbagin and the hydroxyl group of BCDs, as was expected. However, the plumbagin molecule formed two hydrogen bonds between its oxygen atom and two water molecules instead, which could facilitate the formation of an interaction network near BCD cavity. In addition, the enlarged narrow rim of BCD that has been observed earlier occurred because some of the primary hydroxyl groups are bent toward the inner cavity of BCD. Interestingly, even though the plumbagin molecule already migrated out from BCD’s cavity in the BCD-II conformation, it formed several hydrogen bonds with secondary hydroxyl groups and O4 atom of BCD, along with another two water molecules. This interaction should be the consequence of high distortion of BCD structure which is similar to the dynamic motion of BCD in BCD-I conformation. Therefore, this should be the reason that the binding energy of these two inclusion complexes turned out to be stronger than expected.

For MBCD-I conformation, the plumbagin molecule formed two hydrogen bonds between its oxygen atom and two water molecule, which is exactly the same as the interaction found in BCD-I conformations. On the other hand, the plumbagin molecule formed two hydrogen bonds between its oxygen atom and hydroxyl group with O4 atom of MBCD in MBCD-II conformation, which facilitates the binding of plumbagin inside MBCD inner cavity. The visualization also revealed another interesting motion of MBCD which turn out to be similar to BCD. Several methyl groups on narrow rim of MBCD migrated inside the hydrophobic cavity and made the inner cavity of MBCD shallow. Therefore, this could be the reason behind the floating of the plumbagin molecule to the wider rim of MBCD.

As expected, the plumbagin molecule formed several hydrogen bonds between its oxygen atoms with secondary hydroxyl groups and O4 atom of HPBCD, along with water molecules in both conformations. Therefore, this can confirm that the plumbagin molecule tends to stay inside the encapsulated cavity of HPBCD. 

### 2.4. Hydrogen Bonding Lifetime of Inclusion Complexes and the Dynamic of Water Molecules 

**Figure 6:**
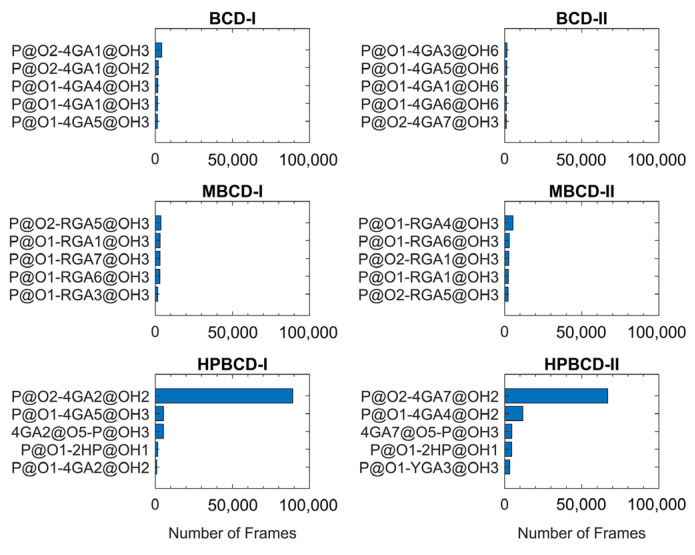
The hydrogen bonding frequency plot of all inclusion complexes with the highest five ranks is shown as blue horizontal bars. Y-axis labels indicate the interacting atom pairs and x-axis labels indicate the number of frames that the hydrogen bonding occurred between each pair. The plumbagin molecule is denoted by P. The hydroxypropyl group is denoted by 2HP. The glucose units are denoted by 4GA, RGA, or YGA. Please note that the number after glucose unit notation refers to the numeric order of glucose units in BCDs. The one and two letter abbreviations of the atom and functional group name, followed by its position in the numeric form, are specified after @ symbol.

The lifetime analysis of hydrogen bonding between BCDs and plumbagin throughout 200 ns MD simulations (200,000 frames) can provide more detailed information about the time-dependent behavior of the hydrogen bonds. The distance and angle cutoff for the hydrogen bond used in the calculation were 3.0 Å and 135°, respectively. The highest five ranks of hydrogen bonding frequency from each inclusion complex are illustrated in Figure 6.

The plots in Figure 6 clearly show that hydrogen bonding between the oxygen atom of the plumbagin molecule and the hydroxyl group from the glucose unit in both HPBCD-I and II conformations had a high frequency. These hydrogen bonds sustained 44.51% and 33.41% of total simulation time in HPBCD-I and HPBCD-II conformation, respectively. Therefore, the frequency of hydrogen bonding between plumbagin molecule and HPBCD was significantly higher than other systems. Moreover, the binding in HPBCD-I conformation tended to be stronger than HPBCD-II conformation, due to its higher degree of sustained hydrogen bonding. Interestingly, the oxygen atom of the plumbagin molecule formed the hydrogen bond with the hydroxyl group of the attached hydroxypropyl chain of HPBCD as well. Even though these bonds were not frequently presented during the simulations, their frequency was considerably higher than some interacting pairs in other systems. Thus, this single hydroxypropyl chain in HPBCD could still be considered as one of the factors that help improve the efficiency of plumbagin binding. 

**Figure 7:**
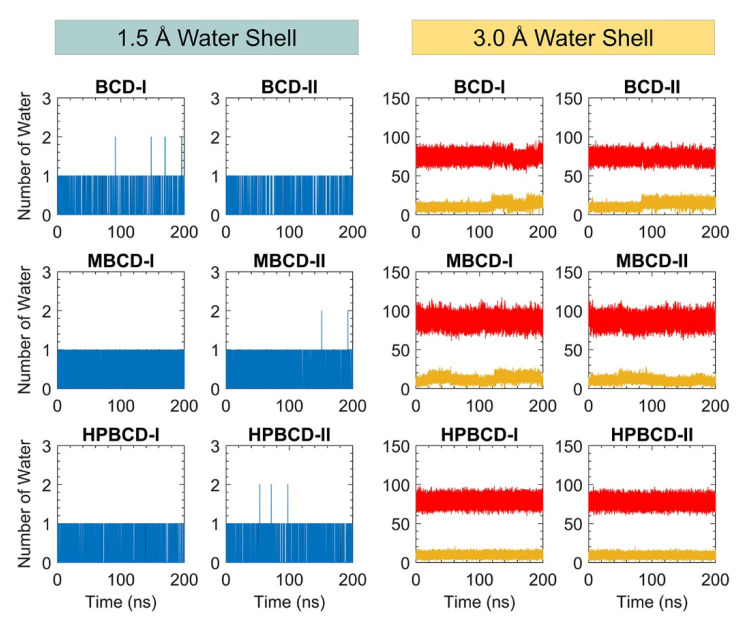
The plot of water molecule counting within defined spherical water shells (1.5 and 3.0 Å) around BCDs and plumbagin molecule throughout 200 ns. The blue and black lines represent the number of water molecules within 1.5 Å water shell around BCDs and plumbagin molecules, respectively. The red and yellow lines represent the number of water molecules within 3.0 Å water shell around BCDs and plumbagin molecules, respectively.

In addition, the dynamic of water molecules around inclusion complexes are investigated to gain more understanding about the role of the water molecule in binding and release states of plumbagin. The water molecule counting around inclusion complexes could represent the dynamic of water molecules and indicate the difference in the water network between binding and release states. However, the total number of water molecules inside the periodic box is large, and distant water molecules should not have significant interaction with the inclusion complexes. Therefore, two layers of spherical water shell were separately defined around BCDs and plumbagin molecules, with a radius of 1.5 and 3.0 Å. The number of water molecules inside both water shells was collected throughout 200 ns simulations, as shown in Figure 7.

There are only one or two water molecules that stayed around BCDs and plumbagin structures within the 1.5 Å water shell, which are shown as blue and black lines in Figure 7. These water counting profiles were consistent with the MD snapshot illustrations in Figure 5, which indicates that the plumbagin molecule interacted with one or two water molecules for all inclusion complexes.

More water molecules were found inside the second water shell with a 3.0 Å radius. The red lines in Figure 7 refer to the number of water molecules around BCDs structure and they are higher than the yellow lines that represent the number of water molecules around the plumbagin. The water molecules counting profiles around BCDs were quite stable, ranging from 60 to 90, 70 to 100, and 65 to 90 molecules for BCD-I/II, MBCD-I/II, and HPBCD-I/II conformations, respectively. The reason that number of water molecules were all stable around BCDs, even though plumbagin molecules migrated out for some systems, was that the hydrophobicity of BCDs inner cavities should not attract more water molecules to fulfill them. 

On the other hand, the water molecules counting profiles around plumbagin are different among inclusion complexes. For BCD-I and BCD-II conformations, the number of water molecules noticeably increased at 120 ns and 90 ns, respectively, which were close to the time that plumbagin leaves the encapsulated cavity. Therefore, the water molecules were attracted by the plumbagin molecule after it migrated from BCD inner cavity. For MBCD-I and MBCD-II conformations, the water molecules counting profiles were the most fluctuated due to the abrupt motion of plumbagin molecule throughout the simulations, as discussed earlier. For HPBCD-I and HPBCD-II conformations, the water molecules counting profiles around plumbagin were very stable, which indicates that plumbagin never left the inner cavity of HPBCD and these were consistent with the results from previous sections.

Consequently, all this information can be used to support the superior stability of plumbagin encapsulation with HPBCD over other BCD derivatives.

## 3. Discussion

The stability analysis of plumbagin–BCDs inclusion complexes, based on all-atom RMSD and distance profiles, suggested that both conformations of plumbagin–HPBCD inclusion complex are the most stable host–guest ligand complex systems. On the other hand, plumbagin molecules tended to migrate from BCD’s inner cavity after some period with a high degree of structural deviation of the BCD molecule. The plumbagin–MBCD inclusion complexes were the least stable systems due to high fluctuation in MBCD structural deviation and the plumbagin molecule was abruptly bounced up and down inside the binding cavity. Moreover, it tended to migrate out of the encapsulate pocket at an early stage of simulation, which indicated the instability of the host–guest complex system.

According to binding energy decomposition, the leading contribution to the binding between plumbagin and BCDs is van der Waals interaction, which is reasonable due to the strong hydrophobicity inside the inner cavity of BCDs. Even though all inclusion complexes have negative binding energy, which indicates the favorable host–guest complexation, it is not necessarily true that the most stable binding will come from the strongest binding energy. Entropy change upon complexation was one important factor that was used for the analysis in this work. BCD-II, BCD-II, MBCD-I, and MBCD-II conformations had positive entropy changes during the latter interval of MD simulations. Thus, these four inclusion complexes tended to be unstable with respect to time.

The intermolecular visualization from the MD snapshots taken during the latter interval of simulation indicated that the plumbagin molecule migrated out from BCD’s cavity. However, it still clings to the outer surface of BCD, with some hydrogen bonding, or forming an interaction network with surrounding water molecules. Despite several interactions presented on the outer surface, these inclusion complexes are not considered to be stable due to the instability of plumbagin inside the shallow inner cavity which occurred from BCD distortion. Similarly, the plumbagin molecule migrated out from MBCD in the MBCD-I conformation and formed an interaction network with water molecules. Even though the plumbagin molecule was still bound inside MBCD’s cavity in the MBCD-II conformation, the stability of this complex system tends to be low due to positive entropy changes and shallow cavity. Inversely, the intermolecular interaction between plumbagin and HPBCD suggested that the plumbagin molecule was well encapsulated within the cavity of HPBCD and it preferred to orient as conformation-I.

In summary, the encapsulation of plumbagin with HPBCD is the most stable. Therefore, HPBCD should be a good candidate for the preservation of plumbagin with longer storage life. Unfortunately, there is no guarantee that plumbagin will migrate out of the inner cavity of HPBCD upon its usage as a medicinal compound. However, the higher temperature inside the human body may play an important role in the release process, and the stable binding between plumbagin molecule and HPBCD could facilitate the slow-releasing mechanism. Therefore, further study on the effect of temperature will be useful to support the development of plumbagin encapsulation for usage as a slow-release drug. We carried out additional simulations for plumbagin–HPBCD complex systems by heating the final configuration from 4 °C (storage temperature) to 25 °C and 37 °C. Then, the systems were equilibrated for 40 ns with similar settings to the simulations at storage temperature. For 25 °C, the plumbagin molecule was well encapsulated as conformation I inside the HPBCD cavity throughout the whole simulation. However, for 37 °C, the alignment of the plumbagin molecule started to change after 20 ns. For HPBCD-I, the plumbagin molecule flipped and aligned as conformation II at 40 ns. For HPBCD-II, the plumbagin molecule pointed its methyl group toward the side of the HPBCD cavity and floated up near the wider rim at 40 ns. This confirmed that a higher temperature, such as body temperature (37 °C), could trigger the release of plumbagin by promoting the less stable molecular alignment.

## 4. Materials and Methods

### 4.1. Plumbagin and BCDs Structures Preparation

**Figure 8:**
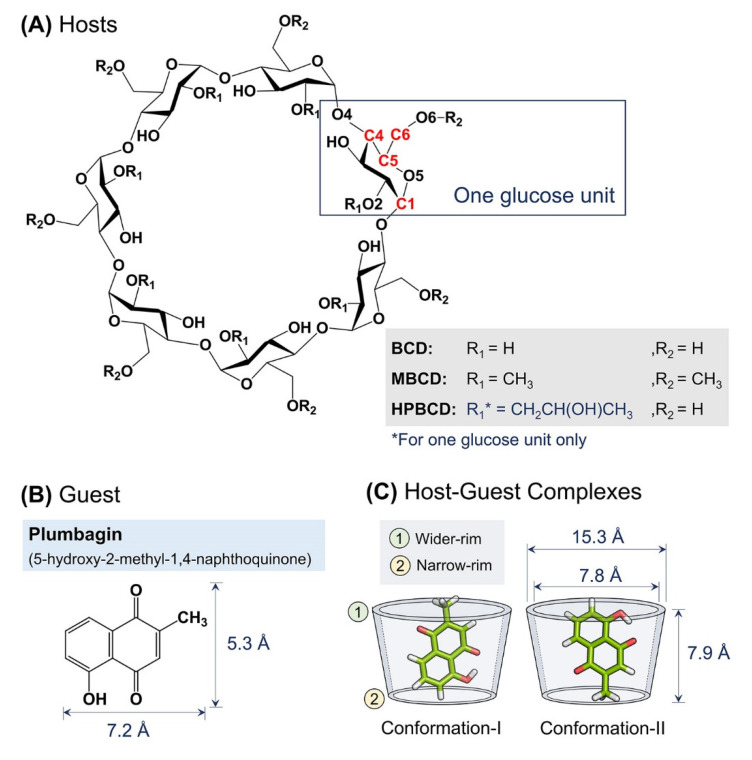
Schematic representations of: (**A**) Glucose unit and atomic numbering of BCD, MBCD, and HPBCD. (**B**) Plumbagin structure and its dimensions. (**C**) Two major conformations of plumbagin–BCDs inclusion complexes and BCD’s dimensions.

The crystalline structure of plumbagin, BCD, MBCD, and HPBCD were downloaded from Cambridge Crystallographic Data Centre [21] with the Cambridge Structural Database (CSD) entry, listed as follows: PVVAQS01 [22], BCDEXD03 [23], BOYFOK04 [24], and KOYYUS [18] (Figure 8A,B). BCD and its derivatives consist of seven glucose units, the hydrophilic outer surface originated from primary and secondary functional groups located on the rims of cyclic-oligosaccharides (Figure 8A) [25]. All cyclodextrins have truncated cone shapes, which comprise a wider rim and a narrow rim, as sketched in Figure 8C. For BCDs, the wider rim is defined by the secondary hydroxyl group attached to C2 and C3 atoms or substituted functional groups attached to O2 atom. The narrow rim is defined by the primary hydroxyl group attached to C6 atom or substituted functional groups attached to O6 atom.

Molecular docking and geometry optimization of plumbagin–BCDs inclusion complexes by semi-empirical quantum mechanical PM6 and PM7 methods were performed with a polarizable continuum model in the previous study. Two major stable conformations of inclusion complexes were selected (Figure 8C) [12]. In conformation-I, the methyl group of plumbagin is pointed toward the wider rim of the host molecule. On the other hand, the methyl group is pointed toward the narrow rim in conformation-II.

### 4.2. Molecular Dynamics Simulations of Inclusion Complexes

We performed molecular dynamics simulations of plumbagin–BCDs inclusion complexes, using the AMBER20 program package [26] for the insightful study of their dynamic behavior. The AMBER program package was selected in this study due to its high efficiency to simulate the dynamic behavior of cyclodextrin over other software [27,28]. The force field parameters for the plumbagin molecule were generated by using antechamber module in the AmberTools20 package [26] with AM1-BCC charge method. The inclusion complexes were solvated in the periodic truncated octahedral box with randomly distributed TIP3P water molecules and the buffer distance between the inclusion complex and periodic box wall of 10 Å. The TIP3P water model was selected because a number of biomolecular force fields were already parameterized in conjunction with this three-sites water model [29]. GLYCAM-06j force field for carbohydrates [30] and general AMBER force field (GAFF2) were used throughout the simulations. The periodic boundary condition was applied to all simulations using the PME method for electrostatic interaction. The cutoff used for Lennard-Jones and Coulomb interactions was 10 Å. To avoid steric effects between water molecules and inclusion complexes, energy minimization was firstly performed with 1000 cycles of steepest descent, followed by 1000 cycles of the conjugate gradient. Afterward, all solvated inclusion complexes were gradually heated from 0 to 277.15 K (4 °C) over 100 ps with the volume held constant. Then, MD simulations were performed to equilibrate the system for 200 ns with 2 fs time steps under isotheral-isobaric ensemble (NPT) at constant storage temperature and pressure of 277.15 K and 1 atm, respectively. Berendsen barostat and Langevin thermostat were used for regulating the pressure and temperature during MD simulations. The SHAKE algorithm was used to constrain all bonds involving hydrogen atoms.

The system energies were extracted from MD trajectories for observation of inclusion complexes equilibration process. All-atom RMSDs of plumbagin and BCDs throughout simulations, with structure after heating to storage temperature as reference, were used to monitor the structural stability. In addition, the distance between the center of mass of plumbagin and BCDs were collected to fully investigate the dynamic behavior of plumbagin inside encapsulated cavities. However, the substituted functional groups of two BCD derivatives were not included in the center of mass calculation. The reason is that the large dynamic motion of methyl groups in MBCD and single hydroxypropyl in HPBCD could lead to asymmetrical BCDs structure.

### 4.3. Binding Energy Calculation

One of the well-known methods for the estimation of binding energy between small ligand and biological macromolecules with economical time is molecular mechanics–generalized born surface area continuum solvation (MM/GBSA) [19]. In this approach, the energy difference (Δ*E*) between host–guest complex and individual free-forms is calculated based on the free energy of three states as follows: free-guest (*E_G_*), free-host (*E_H_*), and inclusion complex (*E_H/G_*).

Δ*E* = *E_H/G_* − (*E_H_ + E_G_*) (1)

The generalized born (GB) model in AMBER uses a sphere to represent each atom in a molecule; additionally, the interior of the atom is assumed to be uniformly filled with a material of low dielectric constant (*ε* = 1). The molecule is surrounded by a solvent of a high dielectric constant (*ε* = 80). The GB model approximates electrostatic energy (*E_gb_*) by a formula as shown below:

*E_gb_* = *E*_*ε*=80_ − *E*_*ε*=1_ (2)

The nonpolar energy (*E_npol_*) is proportional to the total solvent accessible surface area (SA) of the molecule with a constant derived from experimental solvation energies of small non-polar molecules. Then, a fast LCPO algorithm [31] is used to compute an analytical approximation. 

The energies difference term can be categorized into four energetic contributions from two phases. First, the van der Waals (Δ*E_vdw_*) and electrostatic (Δ*E_ele_*) energies contributed to the average interaction energy in gas phase (Δ*G_gas_*), which was calculated according to the same force fields used in MD simulations. Second, the electrostatic (Δ*E_gb_*) and nonpolar (Δ*E_npol_*) energies contributed to solvation-free energy in the implicit aqueous phase (Δ*G_sol_*), which were calculated by using the algorithms mentioned above. Thus, the summation between average interaction energy and solvation-free energy is the total energy difference (Δ*G_Total_*), as follows:

Δ*G_gas_* = Δ*E_vdw_* + Δ*E_ele_* (3)

Δ*G_sol_* = Δ*E_gb_* + Δ*E_npol_* (4)

Δ*G_Total_* = Δ*G_gas_* + Δ*G_sol_* (5)

Lastly, the entropy change (*T*Δ*S*) upon complexation of host and guest molecules at simulated temperature is taken into account to compute the binding energy (Δ*G_bind(MM/GBSA)_*). The entropy calculation has been performed by quasi-harmonic entropy approximation, as follows:

Δ*G_bind(MM/GBSA)_* = Δ*G_Total_* − *T*Δ*S* (6)

## 5. Conclusions

MD simulations of plumbagin–BCDs inclusion complexes under storage temperature (4 °C) revealed that the encapsulation of plumbagin with HPBCD was the most efficient, while MBCD could not produce stable encapsulation. BCD can encapsulate plumbagin inside its inner cavity for some period of time, but its structural distortion also triggers the release of plumbagin similar to MBCD. In addition, the sustained hydrogen bonding between plumbagin molecule inside HPBCD cavity as conformation I tend to promote the superior ability of plumbagin encapsulation. The single hydroxypropyl chain attached to the wider rim of HPBCD may also play an important role to facilitate the binding with plumbagin through weak hydrogen bonding. Therefore, we are convinced that HPBCD should be one of the good candidates as an encapsulation agent for plumbagin, which could support the longer storage life and slow-release mechanism.

## Figures and Tables

**Table 1:** Binding energies of all inclusion complexes, presented with major energetic components in kcal/mol from MM/GBSA calculation during two stable intervals.

Component	BCD-I	BCD-II	MBCD-I	MBCD-II	HPBCD-I	HPBCD-II
15–20 ns interval						
Δ*G_gas_*	−10.11 ± 2.67	−10.61 ± 3.09	−15.83 ± 5.54	−27.72 ± 3.56	−28.93 ± 3.83	−31.32 ± 3.43
Δ*G_sol_*	5.49 ± 1.77	5.92 ± 2.24	4.89 ± 1.64	10.54 ± 2.19	14.77 ± 3.07	15.42 ± 2.65
Δ*G_Total_*	−4.62 ± 1.66	−4.69 ± 1.64	−10.94 ± 4.22	−17.18 ± 2.58	−14.16 ± 1.98	−15.90 ± 2.04
*T*Δ*S*	8.91	7.04	5.37	−0.86	−6.69	−7.98
Δ***G_bind(MM/GBSA)_***	**−13.53**	**−11.73**	**−16.31**	**−16.32**	**−7.47**	**−7.92**
145–150 ns interval						
Δ*G_gas_*	−25.56 ± 2.65	−9.97 ± 3.43	−4.92 ± 5.06	−29.84 ± 3.30	−29.21 ± 3.94	−33.52 ± 4.77
Δ*G_sol_*	11.53 ± 1.93	5.58 ± 2.34	2.01 ± 1.97	11.52 ± 1.83	14.74 ± 3.04	18.22 ± 3.51
Δ*G_Total_*	−14.03 ± 1.74	−4.39 ± 1.93	−2.91 ± 3.39	−18.32 ± 2.41	−14.47 ± 1.93	−15.30 ± 2.31
*T*Δ*S*	9.60	33.14	34.91	0.06	−4.42	−5.54
Δ***G_bind(MM/GBSA)_***	**−23.63**	**−37.53**	**−37.82**	**−18.38**	**−10.05**	**−9.76**

**Table 2:** Comparison of binding energy and total energy difference of plumbagin–BCDs inclusion complexes during the bound state in kcal/mol, with published results from the literature.

Inclusion Complex	Results from This Study		Results from Published Literature	
	Δ*G_Total_*	Δ*G_bind(MM/GBSA)_*	Binding Energy	Calculation Technique
**BCD-I**	−4.62 ± 1.66	−13.53	−5.03 ^a^ [10]	Molecular docking
			−6.18 ^a^ [12]	Semi-empirical PM6
**BCD-II**	−4.69 ± 1.64	−11.73	−5.00 ^a^ [10]	Molecular docking
			−6.15 ^a^ [12]	Semi-empirical PM6
			−4.90 ^a^ [20]	Molecular docking
**MBCD-I**	−10.94 ± 4.22	−16.31	−8.03 ^a^ [12]	Semi-empirical PM6
**MBCD-II**	−17.18 ± 2.58	−16.32	−12.78 ^a^ [12]	Semi-empirical PM6
**HPBCD-I**	−14.16 ± 1.98	−7.47	−9.08 ^a^ [12]	Semi-empirical PM6
**HPBCD-II**	−15.90 ± 2.04	−7.92	−5.70 ^a^ [12]	Semi-empirical PM6

^a^ Binding energy without entropic contribution (exclude entropy change).

## Sample Availability

Samples of the compounds are not available from the authors.
